# Flexible controls of broadband electromagnetic wavefronts with a mechanically programmable metamaterial

**DOI:** 10.1038/s41598-018-38328-2

**Published:** 2019-02-12

**Authors:** Shuo Liu, Lei Zhang, Guo Dong Bai, Tie Jun Cui

**Affiliations:** 1 State Key Laboratory of Millimeter Waves, Southeast University, Nanjing, 210096 China; 2 Synergetic Innovation Center of Wireless Communication Technology, Southeast University, Nanjing, 210096 China

## Abstract

Coding and programmable metamaterials have experienced a rapid development since 2014, leading to many physical phenomena and engineering applications from microwave to terahertz frequencies, and even in the acoustic regime. The major challenge for current programmable metamaterials based on switching diodes is the experimental realization of a huge number of feeding lines for independent control of each digital unit. In this work, we provide an alternative approach for the experimental realization of the programmable metamaterial by developing a mechanical system, which consists of an array of metal blocks with adjustable height. The system supports the combination with conventional coding metamaterials to take full controls of both the phase and polarization of EM waves. As a theoretical byproduct of this work, we propose group delay code to achieve diffraction-limited achromatic redirection of linearly polarized broadband beam from 4 to 6 GHz by combining the group-delay code with the conventional phase code, a feat that traditionally requires complex structural design of unit cell. In view of the multifunctional performance afforded by the full-control of the phase, polarization and group delay, the mechanically controllable metamaterial in the microwave region may benefit different applications, such as imaging, communication, and radar detection.

## Introduction

Coding and programmable metamaterials^1^, a new type of metamaterial described in a direct manner with discretized reflection/refraction phase, have received increasing attention from the metamaterial community since they were initially proposed in 2014. In analogy to the digital circuit, the reflection/refraction phase of coding particles is optimized as 0° and 180° for the 1-bit coding metamaterial, and is divided into 2^n^ equal portions across the 2π phase range for n-bit coding metamaterial. It is worthwhile to mention that the digital description of coding metamaterials allows us to revisit metamaterials from the perspective of information science. For example, based on the Fourier transform between the coding pattern and radiation pattern, we can directly apply many theorems and analytical tools in digital signal processing and information science to the design of coding metamaterials, which have led to many interesting physical phenomena and functionalities^1–5^. There have been great advances in coding and programmable metamaterials in recent year from microwave, terahertz, to acoustic regimes, including anisotropic coding metamaterial^6,7^, frequency-dependent coding metamaterial^8^, acoustic coding metamaterial^9^, free-standing transmission-type coding metamaterial^10^, tensor coding metamaterial^11^, frequency coding metamaterial^12^, Pancharatnam-Berry coding metasurface^13^, etc. Some of them have potential applications in the field of microwave engineering, for example, the reprogrammable coding metasurface hologram for dynamically generating holographic images^14^, the microwave imaging system for imaging object with single-sensor at a single-frequency^15^. Two recent reviews summarized the modeling, design, and attainable physical effects, as well as the prospective applications of coding and programmable metamaterials^16,17^.

Coding metamaterials are compatible with digital logic devices due to its digital representation of the reflection phases makes. By loading one or multiple pin-diodes to the digital particle and switching them on and off, we can dynamically control the reflection/refraction phases. Electrically controlling an array these digital particles with a field programmable gate array (FPGA) via a preset coding pattern (i.e. the preset binary code embedded in FPGA), we can have a real-time manipulation of the incident wave and generate arbitrary radiation patterns to achieve desired functionalities. This is the first generation of the programmable metamaterials, which was initially proposed and experimentally demonstrated at microwave in 2014^1^. Although such an electrical approach possesses the highest modulation speed compared with other approaches (e.g. thermally, optically), issues of sophisticated feeding line layout and complex structure design lay critical roles in practical realizations, especially for higher-bit cases. The limited accessible range of phase and amplitude of electric-controlled programmable metamaterial (ECPM) cause inherent limitation in both device functionality and performance. In addition, they suffer from narrow working bandwidth due to the large chromatic aberrations. Some recent works on reconfigurable metamaterials provide alternative approaches for the realization of programmable metamaterials in the mechanical, thermal and optical manners. Microfluidics structure, as one of the mechanical methods, takes precise control of the metallic fluids in geometrically constrained tubes, enabling us to change the phase of the transmitted microwave via tuning the filling factor of the mercury filled in the split-ring resonator^18^. Some other works propose to alter the physical shape of the reconfigurable unit by utilizing the thermal^19–21^, magnetoelastic^22^ and electroelastic^23^ effects of special materials. It is reported that dielectric photonic metamaterial can be utilized to present a giant nonlinear optical response with an external optomechanical forces^24^. Germanium-antimony-tellurium-based films were employed as a phase-change material to develop a reconfigurable metamaterial in the visible spectrum, with their refractive index being re-written by the direct laser writing technique^25^. Most intriguingly, a new technique in biomedical field called the DNA origami, which folds DNA to create non-arbitrary two- and three-dimensional shapes at the nanoscale, showed great potential in the creation of reconfigurable three-dimensional plasmonic metamolecules^26,27^. More approches on spatially reconfigurable nanomechanical metamaterials can be found in ref. ^28^. Nevertheless, these approaches suffer from either low efficiency or the lack of independent control of every unit. In addition, none of these approaches allows the independent manipulation of the phase distribution and polarization state of the incident wave.

A straightforward approach that avoids these limitations is to employ a mechanical structure as the digital units, which can take direct control of the phase and/or polarization of incident waves. In this work, we present the design and experimental realization of a mechanically controlled programmable metamaterial (MCPM) system, which consists of an array of metal blocks with height automatically controlled according to the given coding pattern. To achieve this goal, we deliberately designd and fabricated a set of mechanical devices and the corresponding control units, which work jontly to allow users to switch coding patterns from the graphic user interface (GUI). As mechanical stability and reliability is critical for the MCPM under harsh environment, we have fully considered the mechanical structure design to keep a high precision control of the block’s height under different code pattern updates and mechanical vibration. One of the benefits of the MCPM system is the ability to combine with another coding pattern that controls the Pancharatnam-Berry phase or the polarization state of EM wave. We will also demonstrate the unique functionality of the MCPM in making broadband reflection with minimized chromatic dispersion, which may have promising applications in broadband microwave focusing, imaging and communication. We should note that a recent work reported a circularly polarized reconfigurable reflectarray antenna consisting of 756 micromotor controlled rotated elements^29^. However, it only works for circularly polarized waves through the rotation of the concentric dual split rings.

## Results

### Mechanical realization

**Figure 1:**
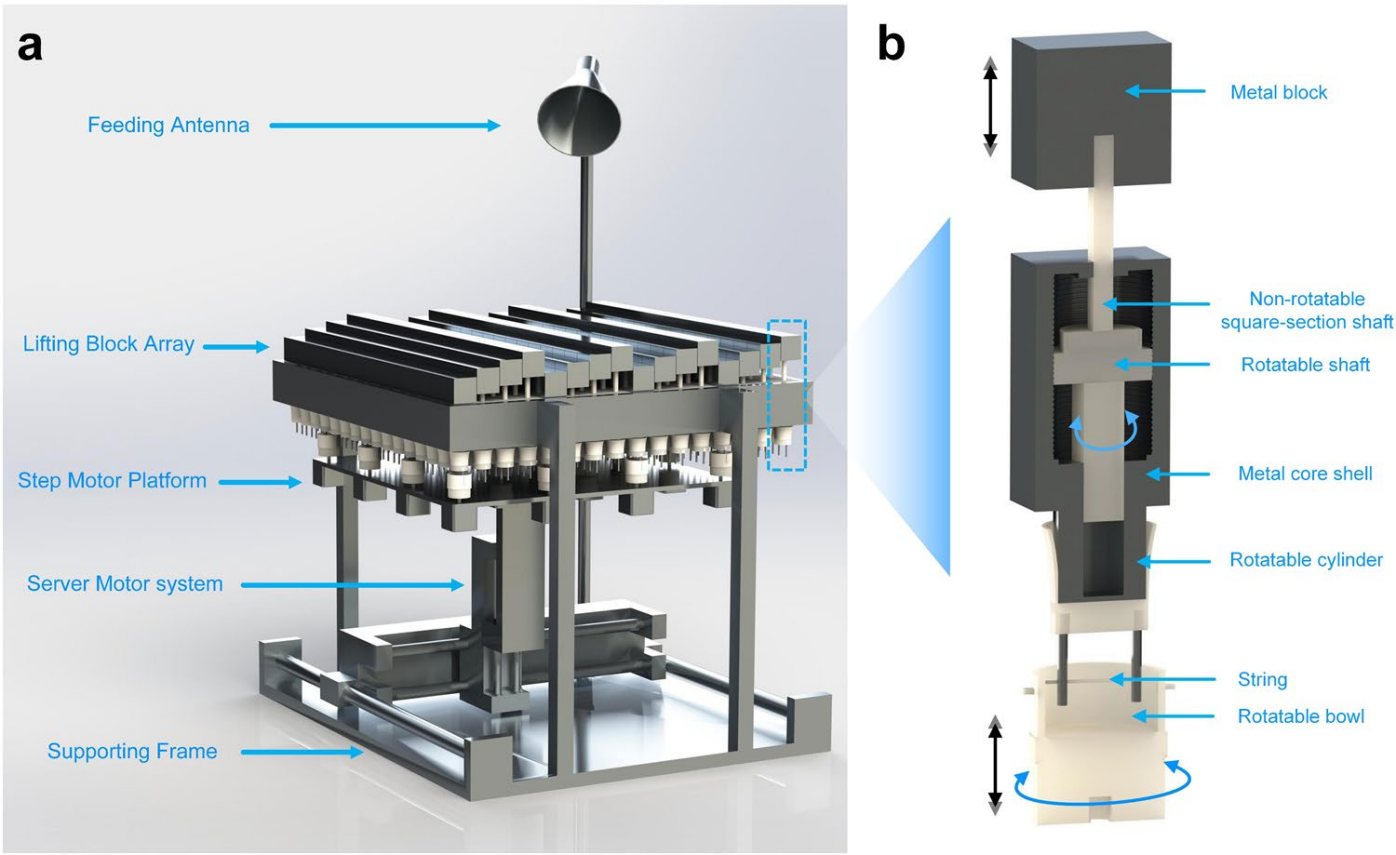
Prototype of the mechanically controlled programmable metamaterial. **(a)** Overview of the entire system. **(b)** Cross-section view of the lifting block array module.

We firstly introduce the MCPM system shown in Fig. 1a, which is built and rendered in a 3D computer-aided design (CAD) design software with real size. The fabricated prototype is shown in Supporting Information Fig. S1. It mainly consist of seven parts from the bottom to the top: a supporting frame, a three-axis server system, a step motor array platform, a lifting block array, a feeding antenna, and a micro control unit (MCU, Supporting Information Fig. S2a). The structure and function of each module are detailed in the method section.

Now, we briefly introduce the working principle of the entire system by describing how these parts work together to achieve the automatic switching of coding pattern. The lifting block device shown in Fig. 1b converts the rotation of the step motor to the accurate movement of metal block in the vertical direction. We employ a 4 × 4 step motor array (Supporting Information Fig. S3) to independently control the height of 256 metal blocks. To realize a switching of a given coding pattern, i.e., to complete the height adjustments of all the metal blocks, the step motor platform needs to move 16 times in the *x* − *y* plane, each time controlling the 4 × 4 metal blocks in their own regions. All the modules work corporately under the control of the MCU (see Supporting Information Fig. S2a), which dynamically acquires the status and controls the position of both the 3-axis server motor system and the 4 × 4 step motor array. Detailed description of each module is provided in the method section. Supporting Information video V1–V6 show the entire process of the MCPM prototype in switching among a series of coding patterns.

Considering the practical implementation of the lifting block module, the lateral dimension of the metal block is optimized as 27.8 mm, with a 0.1 mm gap between each adjacent block to reduce friction. The resonance induced by such a small gap is outside the working frequency range and thus does not affect the phase responses. We should note that due to the fabrication limitation on the mechanical structure of the lifting block array module, it is very challenging to further reduce the physical size down to 1/4 or 1/8 working wavelength, which is, strictly speaking, a requirement for 3D metamaterials to mimic an effective medium with homogenous permittivity (or permeability). However, such a requirement can be relatively relaxed for the design of coding and programmable metasurfaces, where one only consider their reflection/refraction responses. As has been verified from our previous works, the electrical size of the coding particle, which is usually between 1/3 and 1/6 wavelength, has little influence on the wavefront shaping performance. For ease of demonstration, we will take 2-bit MCPM as an example to demonstrate its multifunctional performance. Higher-bit programmable metamaterials can be readily realized with such a mechanical system by reprograming the height of metal block in the MCU. Supporting Information Fig. S7 shows the reflection spectra of the four digital states 00, 01, 10, and 11 in the frequency range from 4 to 6 GHz, obtained by lifting the metal block to the height of 0, 7.5, 15, and 22.5 mm, respectively. Despite the fact that the digital units are designed to work at the central frequency of 5 GHz, they have a flat phase responses across a broad bandwidth, offering them broadband performance superior than the ECPM^1,14,15^. In Supporting Information Fig. S8, we demonstrate the excellent performance of the MCPM in redirecting the normally incident beam to a single or multiple directions with three different coding patterns, where the principle of scattering pattern shift^3^ and addition theorem^5^ are successfully applied to produce arbitrary radiation patterns. It is also noted that although the four digital states are not at the same height, the radiation patterns under both the *x* and *y* polarizations have almost the same performance (Supporting Information Fig. S9), enabling the MCPM to function properly under arbitrary polarizations.

However, this is not the main purpose we develop the MCPM system. In the following, we will demonstrate with three examples how we realize simultaneous control of the wavefront, polarization, and group delay with the combination of two independent coding patterns. This can be practically implemented by attaching conventional coding metamaterial onto the flat surface of the metal block. Note that as the prototype has a dimension of 456 × 552 × 1005 mm with a heavy weight of 55 kg, we are not able to carry out far-field measurements in the microwave chamber. All the results provided are obtained from numerical simulation results in CST Microwave Studio, with sufficient accuracy.

### Simultaneous control of phase and polarization

**Figure 2:**
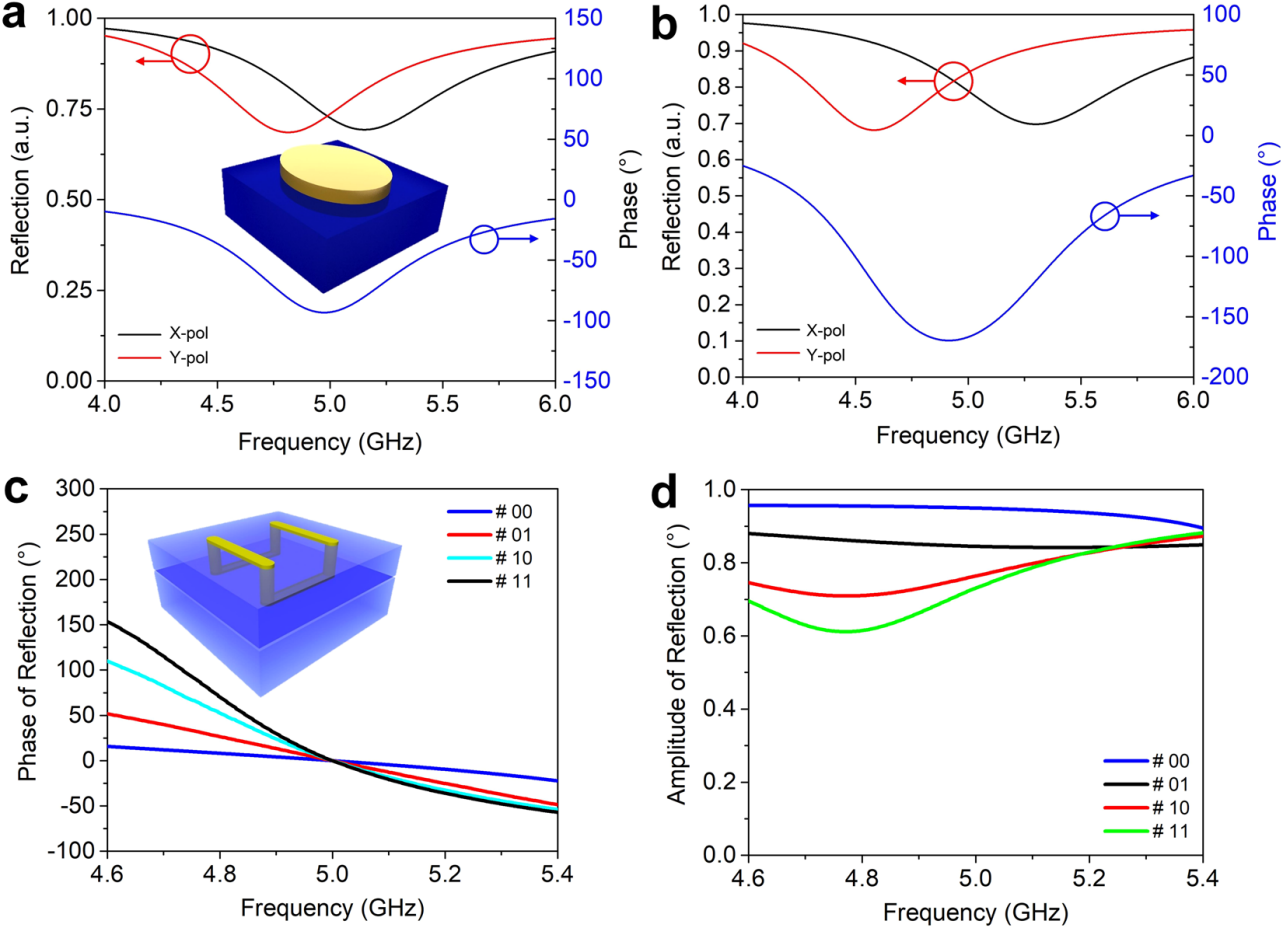
Structures for the anisotropic and group delay coding units. **(a)** Simulated phase difference between the x and y polarizations, and the corresponding reflection amplitudes, for the polarization conversion coding unit shown in the inset. **(b)** Simulated phase difference between the x and y polarizations, and the corresponding reflection amplitudes, for the spin Hall effect coding unit shown in the inset. **(c)** Simulated reflection phases and (**d**) amplitudes for the four group delay coding units.

**Figure 3:**
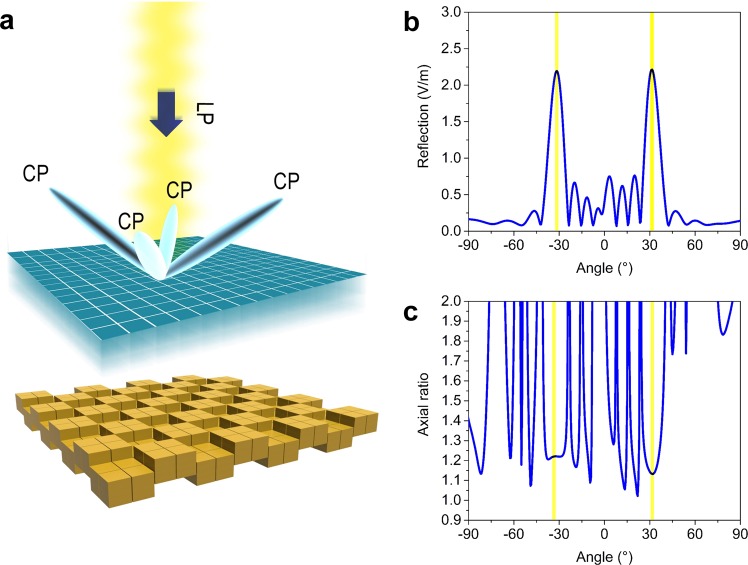
Simulation results of the reflection-type quarter wave plate with arbitrary radiation pattern. **(a)** Schematic illustration of the conversion from the normally linearly polarized beam to four identical beams with circular polarization. The coding patterns of the polarization conversion coding units and mechanical coding units are all zero and chessboard, respectively. **(b)** Simulated 2D radiation pattern in the 45° plane with respect to the *x/y* axis. **(c)** Simulated axial ratio in the in the 45° plane with respect to the *x/y* axis.

The first example demonstrates a reflection-type quarter wave plate with arbitrary radiation patterns. To achieve this goal, we employ the anisotropic structure previously designed in ref. ^7^, as presented in the inset of Fig. 2a, which is composed of an ellipse-shaped metallic disk printed on top of a dielectric substrate (FR4, *ε* = 4.3 + i × 0.129). Detailed dimensions are given in Supporting Information Fig. S6a. The simulated phase difference between the *x* and *y* polarizations are plotted in Fig. 2a, which reaches 90° at the designed frequency 5 GHz. The amplitude of reflection for both polarizations equals 0.7 at 5.0 GHz, making the ellipse-shaped structure an ideal linear-to-circular polarizer. If we attach the ellipse-shaped structure onto all the metal blocks of MCPM with chessboard coding pattern, as schematically illustrated in Fig. 3a, we can convert the linearly polarized normally incident beam to four equal beams with circular polarization. As we have assumed periodic boundary condition in the unit cell simulation, where the near-field coupling effect between adjacent unit cells having different geometries is not considered, super unit cell consisting of an array of identical unit cells are preferred to be used in the coding pattern design^1–3^. Figure 3b shows the simulated far-field radiation pattern in the vertical plane 45° with respect to the *x/y* axis, where two obvious beams can be observed at the elevation angle of ± 32°. We further look into the axial ratio of the reflected beam in the same plane as shown in Fig. 3c, which ranges from 1.1 to 1.2 in the main beam directions, indicating an ideal circular polarization.

**Figure 4:**
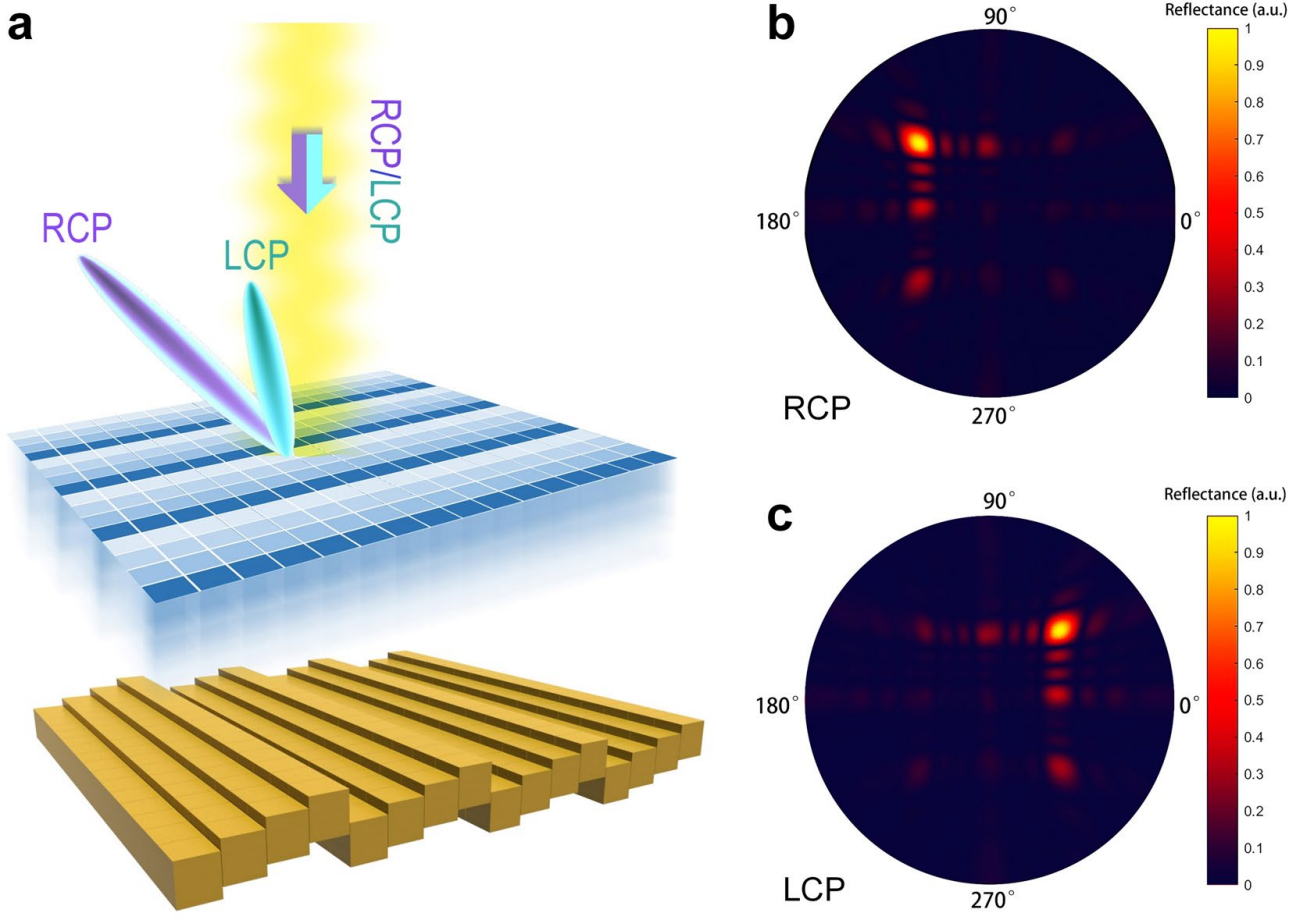
Splitting of RCP/LCP waves realized with the combination of phase coding pattern and PB phase coding pattern. **(a)** Schematic illustration of the splitting of RCP/LCP waves, in analogy to the SHE effect. The RCP/LCP beam are reflected to different directions. The coding patterns of the PB coding units and mechanical coding units are both “00 01 10 11 00 01 10 11…” but varying along orthogonal directions. **(b,c)** Simulated 3D radiation patterns under the RCP and LCP illuminations, respectively. Note that the coding sequence for the mechanical coding units indicates the height position 0, 7.5, 15, and 22.5 mm of the metal block, while it implies different unit cells of the PB coding units when the ellipse-shaped disk is rotated along the z-axis by 0°, 45°, 90°, and 135°.

In the second case, we demonstrate the splitting of right-circular polarization (RCP, purple color) and left-circular polarization (LCP, blue color) into two beams in opposite directions, which is in analogy to the spin Hall effect (SHE) observed in quantum physics. As schematically illustrated in Fig. 4a, this functionality is realized by adding a Pancharatnam-Berry (PB) phase coding pattern (indicated by the gradient blue mesh grid) to a normal phase coding pattern of MCPM. The ellipse- shaped structure is again employed as the basic element for the coding metamaterial generating PB phase, with phase difference reaching nearly 180° at 5 GHz and equal amplitudes of around 0.81 under the *x* and *y* polarizations (see Fig. 2b). By rotating the ellipse-shaped disk along the *z*-axis by 0°, 45°, 90°, and 135°, we obtain the four coding units 00, 01, 10, and 11 for the PB phase coding metamaterial, respectively, each having a 90° phase difference to the next one under the RCP/LCP incidence. Stacking such PB phase coding units onto MCPM with the same gradient coding pattern “01230123…”, we obtain a single beam radiation in the direction 135°/49.2° (azimuthal/elevation) under the RCP normal incidence, and a single beam radiation in the direction 45°/49.2° under the LCP normal incidence. With the aid of MCPM, we can redirect the RCP and LCP beams to arbitrary directions by changing the coding pattern of the metal block, which cannot be realized with ECPM.

### Broadband anomalous reflection

All previous works on coding and programmable metamaterials focus only on the beam control at a single frequency point, which inevitably results in undesired performances for broadband signals, including the deviation of the reflection angle or the shift of focus. Such performance deteriorations are caused by the chromatic dispersion, a phenomenon that is observed from the splitting of white light into a rainbow spectrum when it passes through a dispersive prism. Chromatic dispersion should always be avoided in many applications such as communication, imaging, and radar detection where broadband signals are needed. A few works have been recently reported on the alleviation of the chromatic aberrations by exploiting geometric phase combining with phase compensation^30^, engineering multiple resonances into compound broadband performance^31–33^, or stacking multiple layers of resonators with different resonant frequencies^34^. However, they either work for only circular polarization, or require careful optimization.

In the third example, we propose the method for realizing broadband achromatic reflection for linearly polarized wave with a much simpler approach, which is to combine a phase coding pattern with a group delay coding pattern. To explain why the direction of reflection can be kept stable under the broadband illumination with different frequency components, let us first look at the phase distribution required for the broadband anomalous reflection at arbitrary frequency,

1 $$\phi (r,\omega )=-\frac{\omega }{c}\cdot r\cdot \,\sin \,\theta $$

where *ω, c, r, θ* are the angular frequency, light speed, coordinate, and reflection angle, respectively. It is clear from Eq. (1) that the phase distribution *φ(r, ω)* is dependent on both the spatial coordinate and frequency, implying that the phase profile should vary as a function of these two parameters to guarantee a perfect anomalous reflection with fixed angle at different frequencies. We will further investigate Eq. (1) by expanding it with Taylor series near the central frequency *ω*_*0*_ as,

2 $$\phi (r,\,\omega )=\phi (r,\,{\omega }_{0})+{\frac{\partial \phi (r,\omega )}{\partial \omega }|}_{\omega ={\omega }_{0}}(\omega -{\omega }_{0})+\phi (r,\,{\omega }_{0})+{\frac{{\partial }^{2}\phi (r,\omega )}{2\partial {\omega }^{2}}|}_{\omega ={\omega }_{0}}{(\omega -{\omega }_{0})}^{2}+\mathrm{...}$$

In previous works on the coding pattern design of programmable metamaterials, we only focus on the zero-order term in Eq. (2), which is not dependent on frequency. However, for the functionality of anomalous reflection as given in Eq. (1), both the zero-order term $\phi (r,\omega )=-\,\frac{\omega }{c}\cdot r\cdot \,\sin \,\theta$, and the first-order derivative term $\phi ^{\prime} (r,\omega )=-\,\frac{1}{c}\cdot r\cdot \,\sin \,\theta$ should be considered to keep the reflection angle stable in a certain bandwidth around *ω*_*0*_. Higher-order derivative terms are also needed to be considered if the phase profile is dependent on the higher-order exponent of *ω*.

To realize a broadband anomalous reflection with the proposed MCPM, we firstly introduce the concept of group delay code, which corresponds to the first-order derivative of the phase profile that compensates for the time difference of the wavepacket with different frequency components. As a proof of principle, we take the 2-bit group delay code set (00, 01, 10, 11) as an example to demonstrate how it is combined with the phase code of the block array to realize a constant reflection angle under broadband beam illumination of 4-6 GHz. The inset of Fig. 2c shows the structure of the coding element that controls the group delay of wave. It is a dual-layer structure which consists of two pairs of parallel metal wires printed on two dielectric layers, each pair connected through two metallic vias. Detailed dimensions are provided in Supporting Information Fig. S6b and Table 1.

**Table 1:** Geometrical parameters of the group delay coding elements.

Coding unit	*L* (mm)	*a* (mm)	*d*_*1*_ (mm)	*d*_*2*_ (mm)
00	28	10	7	9
01	28	10	3	5
10	28	13	7	3
11	28	10	7	3

The group delay coding unit should be optimized to provide frequency- independent time delay within the frequency band of interest. Figure 2c gives the simulated reflection phases of the four group delay coding elements. For a convenient observation of the slope of the four phase curves, they have been shifted to coincide at the designed frequency 5 GHz. By carefully optimizing the geometrical parameters, we obtain the four group delay elements 00, 01, 10, 11, corresponding to the group delays of 0.13, 0.34, 0.55, 0.73 μs at 5 GHz, respectively, which are calculated in the frequency range of 4.6–5.4 GHz using $\frac{\partial \phi (r,\omega )}{\partial \omega }$. The amplitude of reflection for the four elements ranges from 0.6 to 0.95 in the considered bandwidth, as are shown in Fig. 2d.

**Figure 5:**
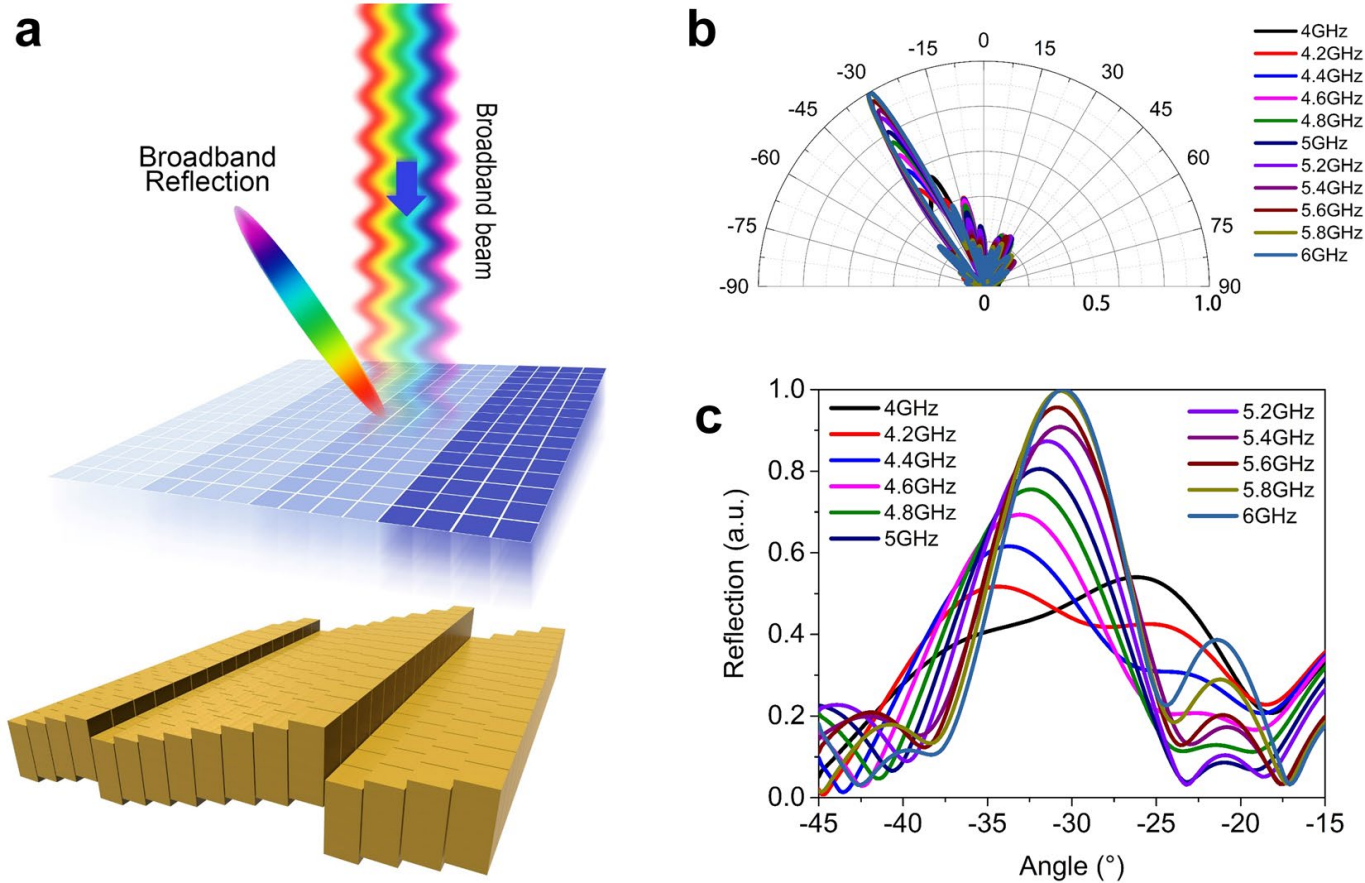
Simulation results of the broadband reflection realized with the combination of phase coding pattern and group delay coding pattern. **(a)** Schematic illustration of the broadband reflection under the illumination of a Gaussian beam modulated at 4–6 GHz. The coding patterns of the group delay coding units and mechanical coding units are “00 00 00 00 01 01 01 01 10 10 10 10 11 11 11 11…” and “00 01 10 11 00 01 10 11…”, respectively. **(b,c)** Simulated 2D radiation pattern for the radiation patterns from 4 to 6 GHz plotted in the polar and Cartesian coordinate. Note that the coding sequence for the mechanical coding units indicates the height position 22.57, 38.56, 6.23, and 0 mm of the metal block, while it implies different unit cells of the group delay coding units listed in Table 1.

Before we start to superimpose the group delay code with phase code, i.e. to attach the group delay coding element to the metal blocks, we need to calculate the height shift of the metal block to compensate the phase difference of the four group delay elements. By setting the initial height of the metal blocks at 22.57, 38.56, 6.23, and 0 mm, we can shift the phase curves of the group delay elements to 0° at 5 GHz, as is shown in Fig. 2c. Combining a phase coding pattern “00 01 10 11 00 01 10 11 00 01 10 11 00 01 10 11” with a group delay coding pattern “00 00 00 00 01 01 01 01 10 10 10 10 11 11 11 11”, as illustrated in Fig. 5a, we are able to redirect a normally incident broadband beam to the direction of 32.4° with minimized chromatic dispersion. The 2D simulated radiation patterns in the plane of interest are plotted in Fig. 5b from 4 to 6 GHz with a step of 0.2 GHz. As expected, the system deflects the incident beam by an angle of ~32.4° (see Fig. 5b,c), as compared to the case without the group delay coding pattern (Supporting Information Fig. S10), where the beam angle ranges from 41° to 27.2° as frequency sweeps from 4 to 6 GHz, which is significantly larger than the compensated case due to the chromatic dispersion. Supporting Information video V7 shows time evolution of the simulated electric field distribution when a Gaussian broadband beam modulated between 4-6 GHz incidents on the MCPM and reflects back to the direction of 32.4°. The slight deviation of the deflection angle from 31.2° to 35° (see Fig. 5c), as is also observed from the non-ideal field distribution, is likely to result from the height difference among different blocks, which is not considered in the unit cell simulation with periodic boundary condition. Near-field coupling between adjacent group delay coding elements having different geometries may also contribute to such unexpected deviation. Thanks to the separation of the phase control and group delay control enabled by this approach, we could also realize achromatic lens under one polarization and hyper dispersion lens under the orthogonal polarization by designing an anisotropic group delay coding unit^34^.

## Conclusion

To conclude, we proposed and realized a mechanically controlled programmable metamaterial system to take full control of the reflected beam in both the phase distribution and polarization state. To achieve this goal, we developed a set of mechanical devices which work jointly under the control of an MCU to allow an independent adjustment of the height of each metal block in the lifting block array. The system can automatically complete the coding pattern update according to the user’s choice from a series of preset coding patterns in the system. With the aid of disk sensor that records the status of every step motor in real time, the system knows the actual height of each metal block and can therefore resume after system interruption, and be able to switch to another coding pattern from arbitrary coding pattern. One of the advantages of MCPM, unless coding pattern update is needed, the maintaining of coding pattern does not consume electricity, which is not the case for ECPM, where the digital particle resets to the initial state as power goes off. Another benefit of MCPM is its ideal EM responses, with almost unity reflection amplitude and 360° phase coverage. Most importantly, the simple wavefront control allows us to combine it with another independent coding pattern, for example, polarization coding pattern, to take full control of both the phase and polarization simultaneously. Of particular interest for the present study is the development of group delay coding, which are combined with the phase coding pattern to realize the reflection of broadband microwave beam to a constant angle with minimized chromatic dispersion.

However, the system proposed here is a proof of concept, not attempting to optimize the size and weight. Additionally, stability and lifetime under ambient or even harsh conditions are critical in practical applications. While there are still some drawbacks of the mechanically programmable metamaterial, it is no doubt that the unique features of it will secure its place in future applications. Future work will, we believe, focus on the further development of new mechanical structure with smaller size and light weight, as well as convenient integration with conventional metamaterials, pushing it towards realistic applications^35^.

## Method

### Each module of mechanical structure

The supporting frame is designed to support all the other modules including the server motor system, the step motor platform, the lifting block array (Supporting Information Fig. S4) and the feeding antenna. The server motor system, driven by the stepper driver (Supporting Information Fig. S2c) and the MCU (Supporting Information Fig. S2a), can move in the *x, y, z* directions with high precision (position error < 0.1 mm). The step motor platform (Supporting Information Fig. S1) holds the 4 × 4 step motor array, with each motor independently controlled by the MCU and a driver (Supporting Information Fig. S2d) to rotate with a precision of ± 0.1 degree. To allow an independent control of the height of each metal block, a mechanical device shown in Fig. 1b is designed, which is mainly composed of a metal block in connection with a non-rotatable square-section shaft, a rotatable shaft that can rotate freely along the screw thread etched on the inner side of the metal core shell, a rotatable cylinder in connection with the rotatable shaft and two metal needles, a rotatable bowl with a soft string. A horn antenna working from 4 to 6 GHz is installed to provide off-axis illumination for the lifting block array. All the metal parts (gray color) are fabricated with Computerized Numerical Control (CNC) machining process, while the non-metallic parts (white color) are prepared using 3-D printing technology with photosensitive resin, considering the lower fraction between plastic and lower cost of complex mechanical structures.

### Working Mechanism

We now explain how each part in the lifting block device (Fig. 1b) works jointly to enable the accurate movement of metal block in the vertical direction. The polyester rotatable bowl, connected to the step motor at the bottom, rotates the rotatable cylinder through the forces between the soft string and two metal needles. The reason for why we use soft string instead of rigid needles is because it is difficult to guarantee a perfect contact between the two vertical needles and the horizontal needles, thus causing overload and even locked rotor to the step motor. The rotatable shaft driven by the rotation of the rotatable cylinder rotates along the screw thread, which leads to a movement in the vertical direction, and pushes/pulls the non-rotatable square-section shaft together with the metal block on the top through a soft joining mechanical structure (not shown in the figure) designed between the non-rotatable square-section shaft and the rotatable shaft. In this way, the metal block can be lifted up/down by clockwise/counter-clockwise rotating the rotatable cylinder. As the rotatable shaft is tightly restricted by the skew thread, it can bear an external force of over 100 N (either pushing or pulling), helping keep the height of the metal block stable under mechanical vibrations. Such a delicate mechanical design also protects the step motor from being damaged by external forces imposed on the metal block, improving the system reliability under harsh environment.

Next, we introduce how we utilize the 4 × 4 step motor array to independently control the height of 256 metal blocks. Considering the size and cost of step motors, it is not practical to incorporate 16 × 16 of them on the step motor platform. As an alternative, we propose to efficiently control the 256 metal blocks using an array of 4 × 4 step motors with a spacing of four metal blocks, as shown in Supporting Information Fig. S3. In this configuration, as each motor is responsible for the 4 × 4 metal blocks in their own regions, the step motor platform needs to move 16 times to complete the height adjustments of all the metal blocks, i.e., one update of coding pattern. In each time, the step motor platform will firstly lifted to the height of rotatable bowl to drive the rotatable cylinder, complete the height adjustment of the current metal blocks, and then descend to the lowest position, followed by a movement in the *x-y* direction to the position of the next metal block, ready for the height adjustment of the next metal block Supporting Information Fig. S5 shows the schematic of the above process.

To let the all the modules work corporately to complete the coding pattern update, a micro control unit (see Supporting Information Fig. S2a) is designed to enable the position control of the server motor system and the precise rotation of the step motor. For example, the MCU dynamically acquires the height of the metal block by real-time recording the number of turns the motor rotates through 16 channels of disk sensors installed besides each step motor. Such a feedback system allows the MCPM to switch to any coding pattern without resetting to the initial status (all-zero coding pattern), even after an unexpected interruption such as mechanical breakdown or power off. To provide users with a convenient update of coding pattern, we have embedded several preset coding patterns into the MCU, which can be executed by simply following the graphic user interface (GUI) on the screen (Supporting Information Fig. S2b). Supporting Information video V1–V6 shows the entire process of the MCPM prototype in switching among a series of coding patterns (Supporting Information Fig. S11).

## Supplementary information

Supporting Information

Video V1

Video V2

Video V3

Video V4

Video V5

Video V6

Video V7
