# Supplementary material for: Flexible controls of broadband electromagnetic wavefronts with a mechanically programmable metamaterial
Source: Sci Rep. 2019 Feb 12;9:1809. doi: 10.1038/s41598-018-38328-2 (PMC6372690; doi:10.1038/s41598-018-38328-2)
Supplement: Supplementary file 1 — Supporting Information [file 41598_2018_38328_MOESM1_ESM.docx]

**Supporting Information Materials for**

**Flexible controls of broadband electromagnetic wavefronts with a mechanically programmable metamaterial**

Shuo Liu^1,2,†^, Lei Zhang^1,2,†^, Guo Dong Bai^1,2^, and Tie Jun Cui^1,2,*^

^1^State Key Laboratory of Millimeter Waves, Southeast University, Nanjing 210096, China

^2^Synergetic Innovation Center of Wireless Communication Technology, Southeast University, Nanjing 210096, China

† These authors contributed equally to this work.

* Corresponding author: [tjcui@seu.edu.cn](mailto:tjcui@seu.edu.cn)

**This PDF file includes:**

Supporting Information Figures S1 to S11


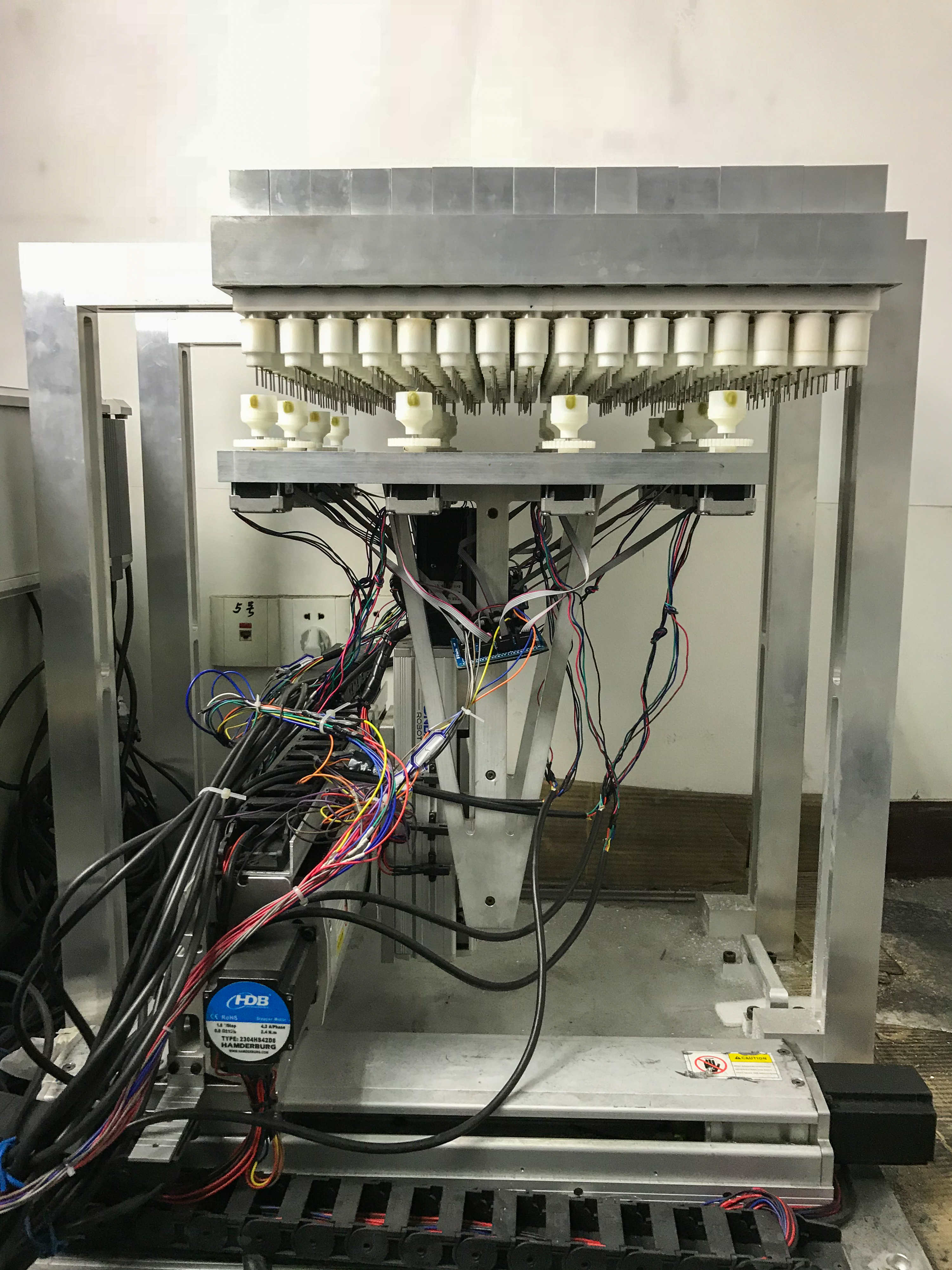


**Supporting Information Figure S1** Photo of the fabricated prototype of the mechanically controlled programmable metamaterial.


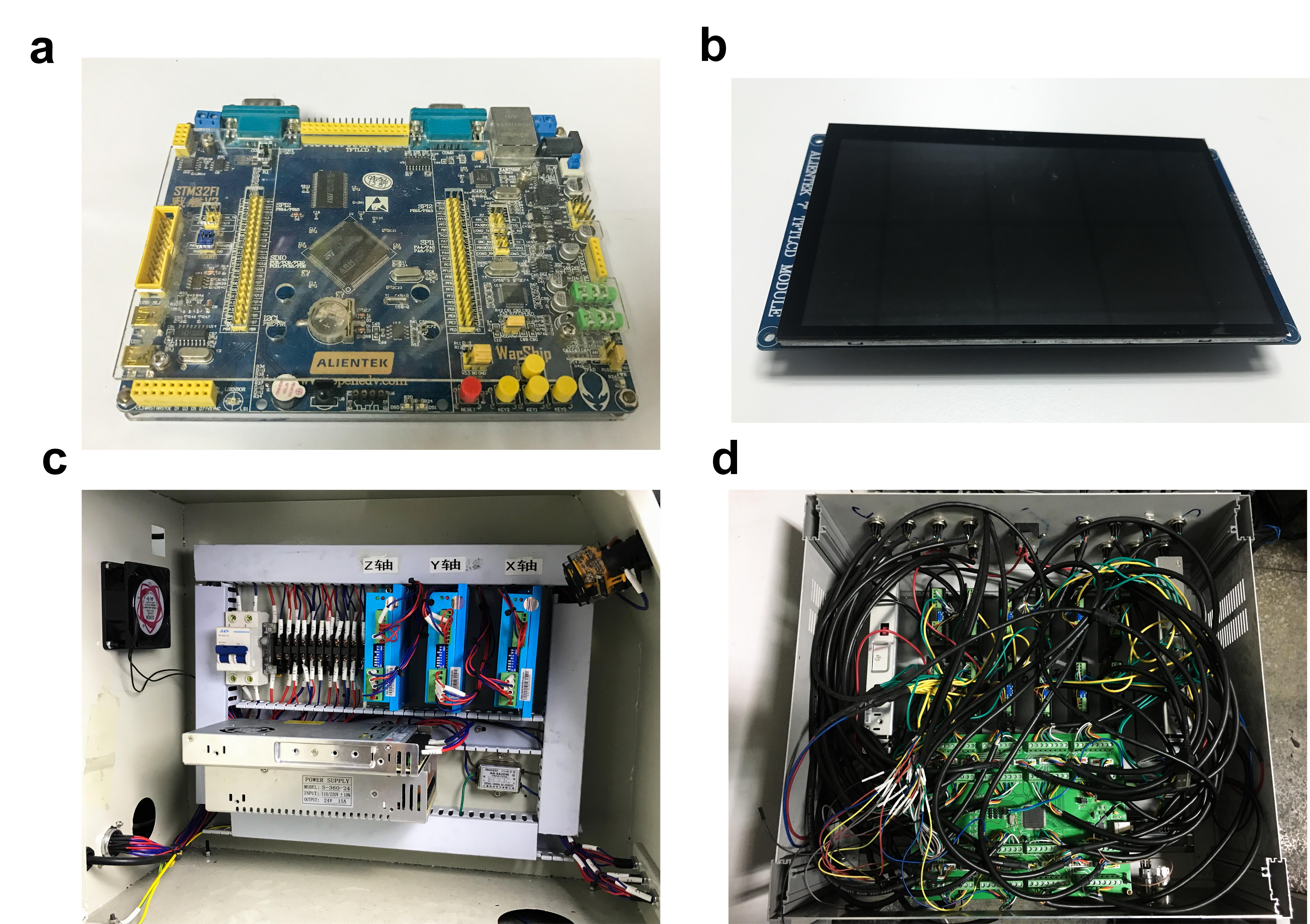


**Supporting Information Figure S2** Part of the prototype of the mechanically controlled programmable metamaterial. a) Micro-control unit. b) Operation screen with graphic user interface. c) Stepper driver for the 3-axis server system. d) Stepper driver for the 16 step motors.


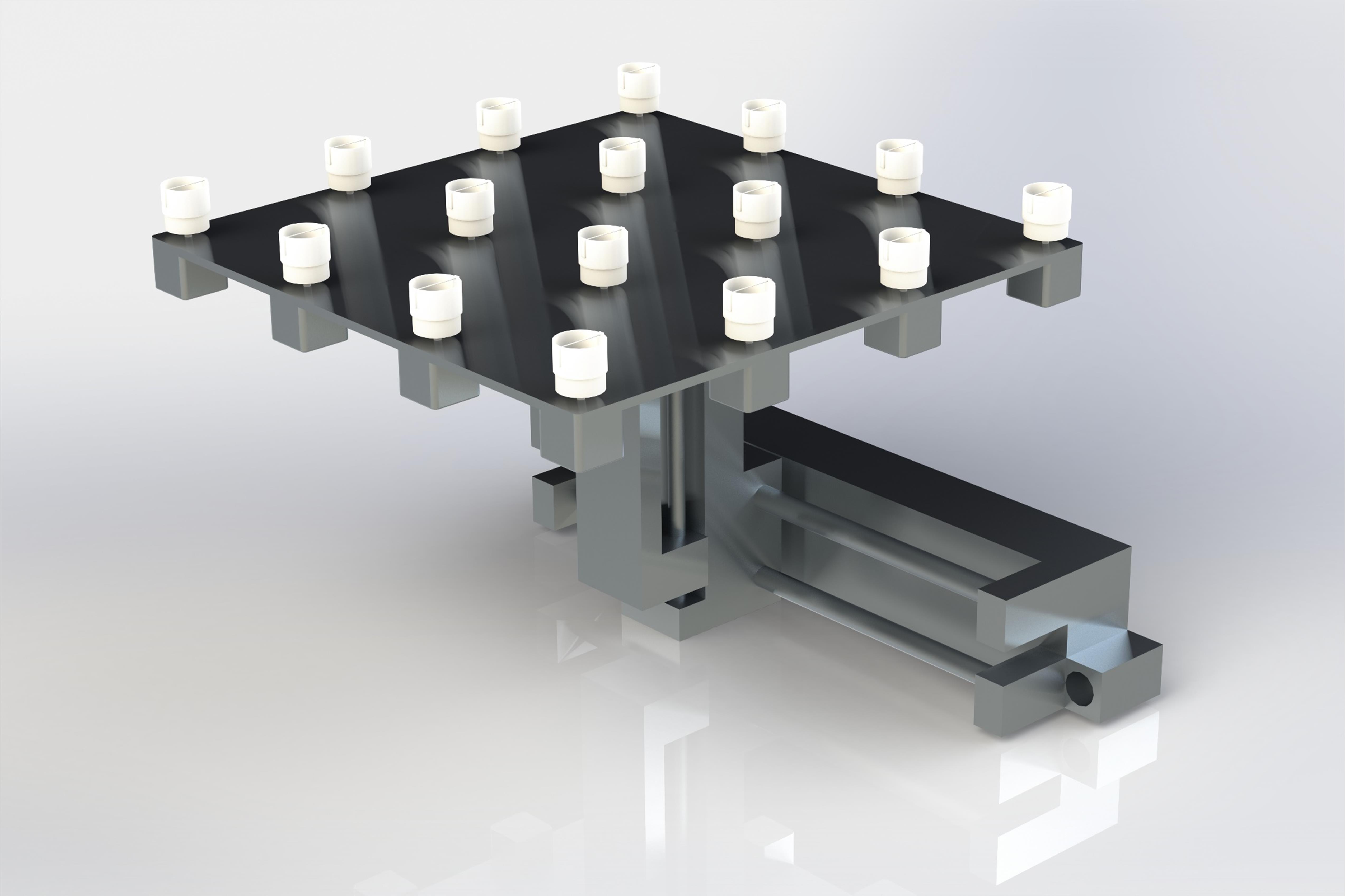


**Supporting Information Figure S3** Step motor platform holding an array of 4×4 step motors.


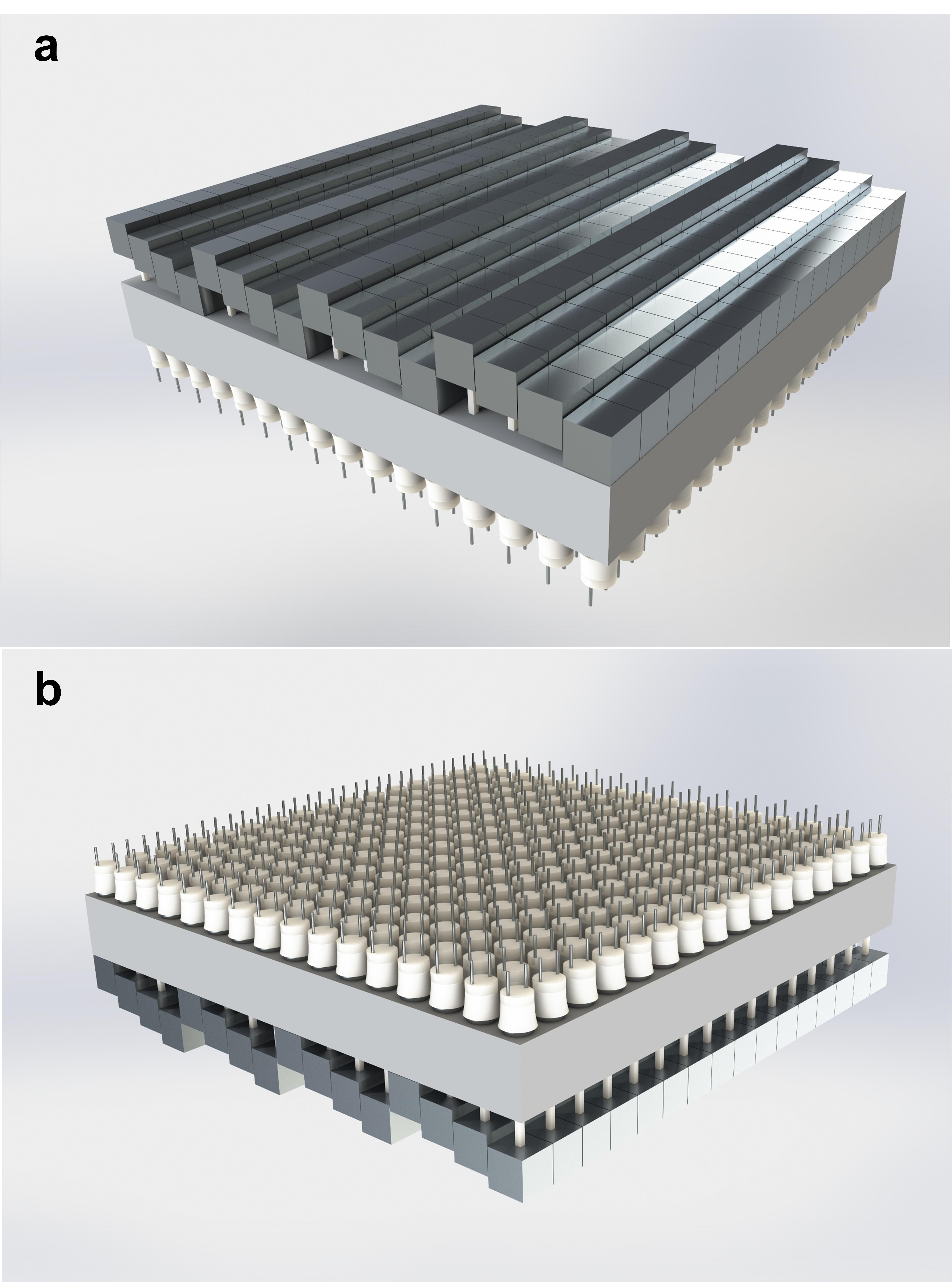


**Supporting Information Figure S4** Lifting block array with 16×16 metal blocks. a) Top view. b) Bottom view.


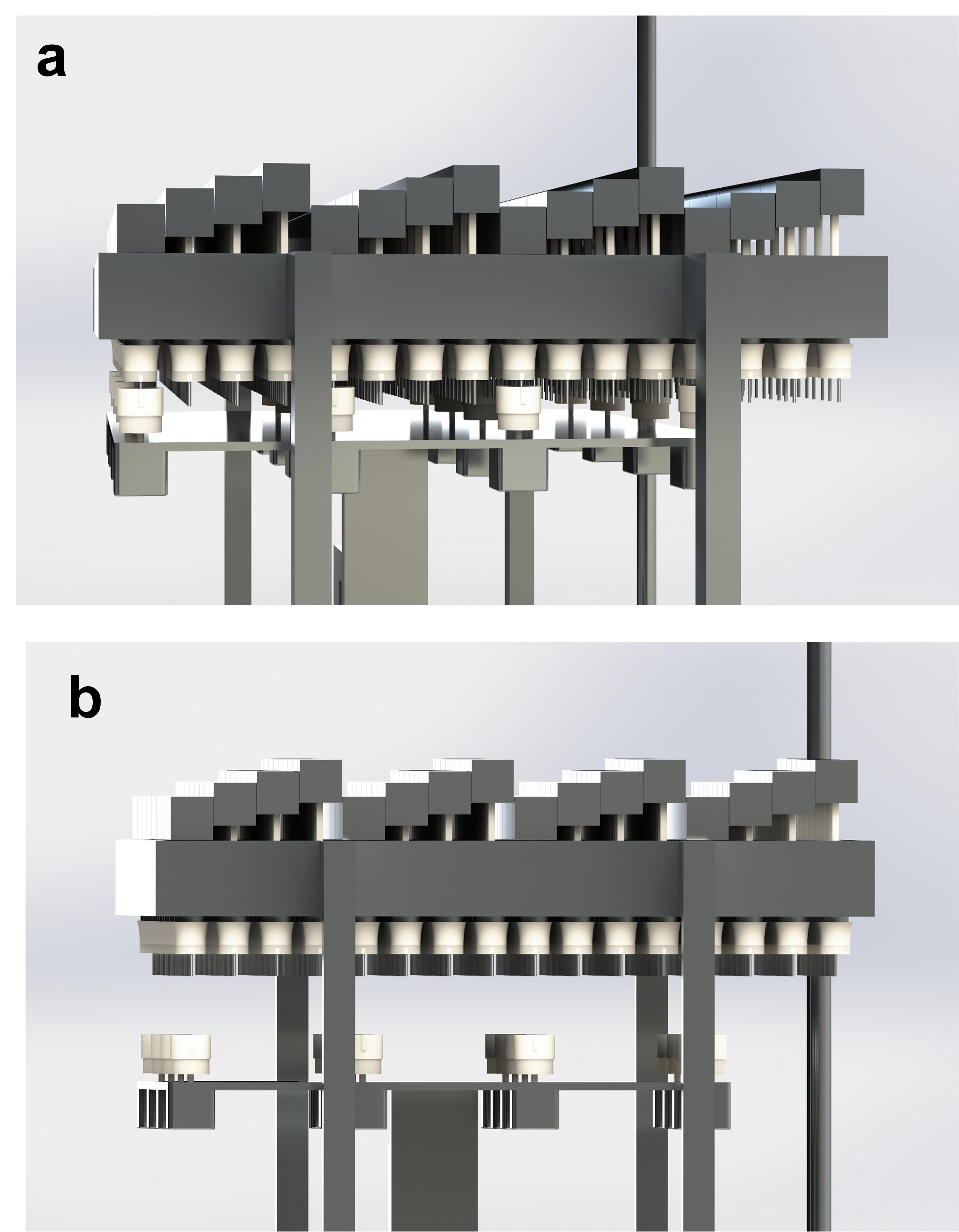


**Supporting Information Figure S5** Schematic illustration of the status when the step motor platform is lifted a) up and b) down.


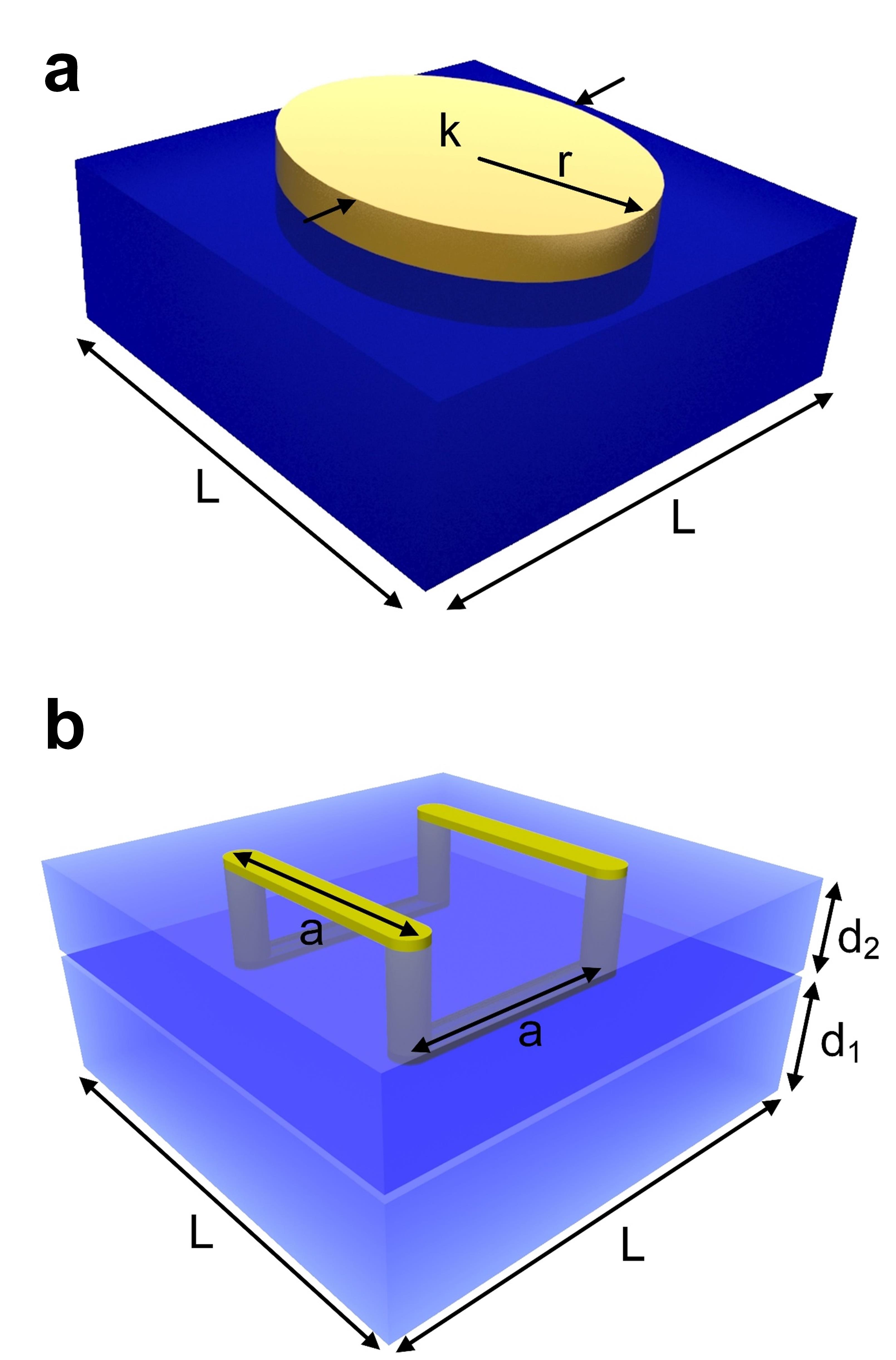


**Supporting Information Figure S6** Structure of the coding unit for the realization of a) reflection-type quarter wave plate and splitter of RCP/LCP, and b) broadband anomalous reflection.


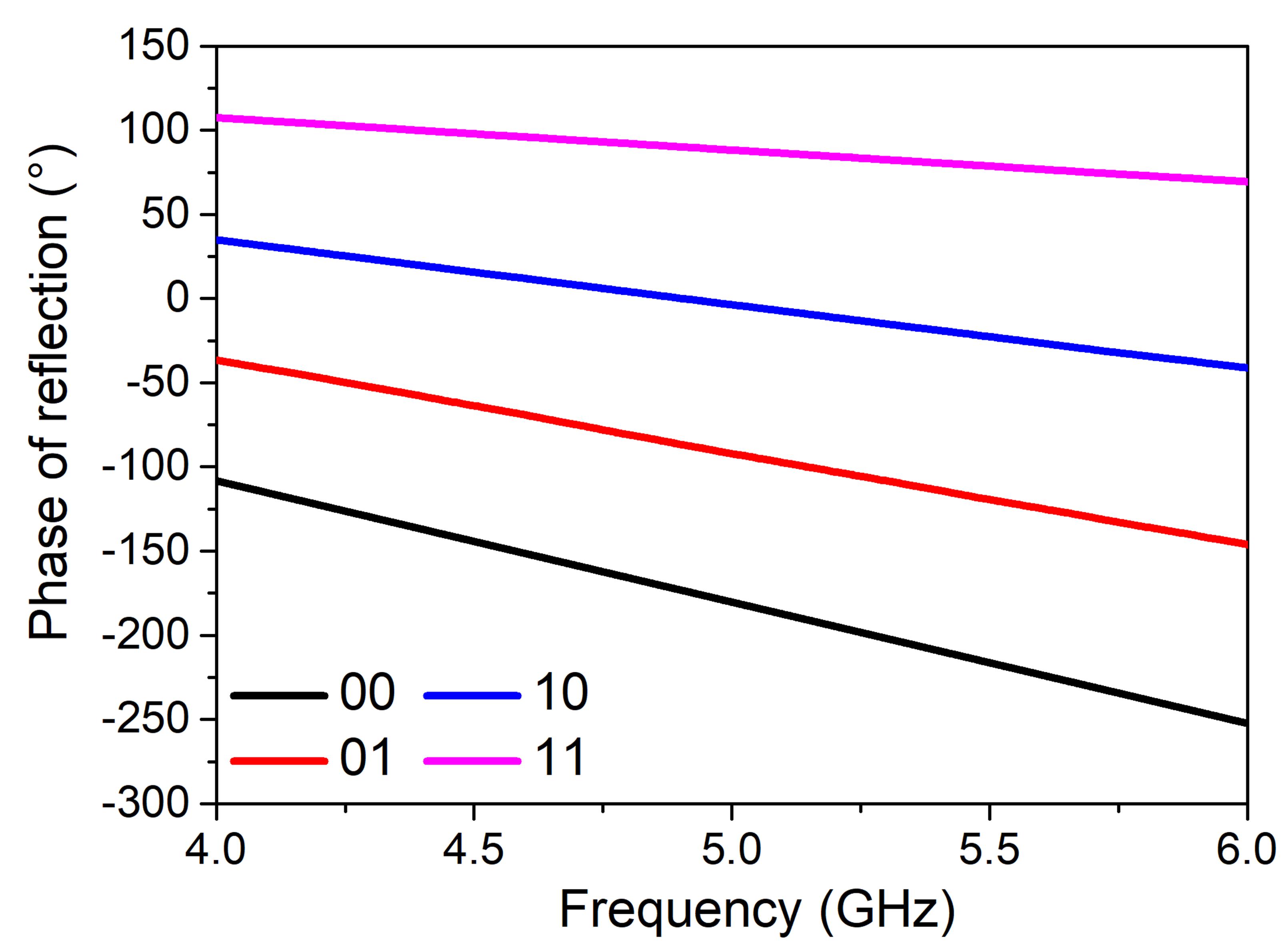


**Supporting Information Figure S7** Simulated reflection spectra of the four digital states 00, 01, 10, and 11 in the frequency range from 4 to 6 GHz, corresponding to the height of metal block of 0, 7.5, 15, and 22.5mm, respectively.


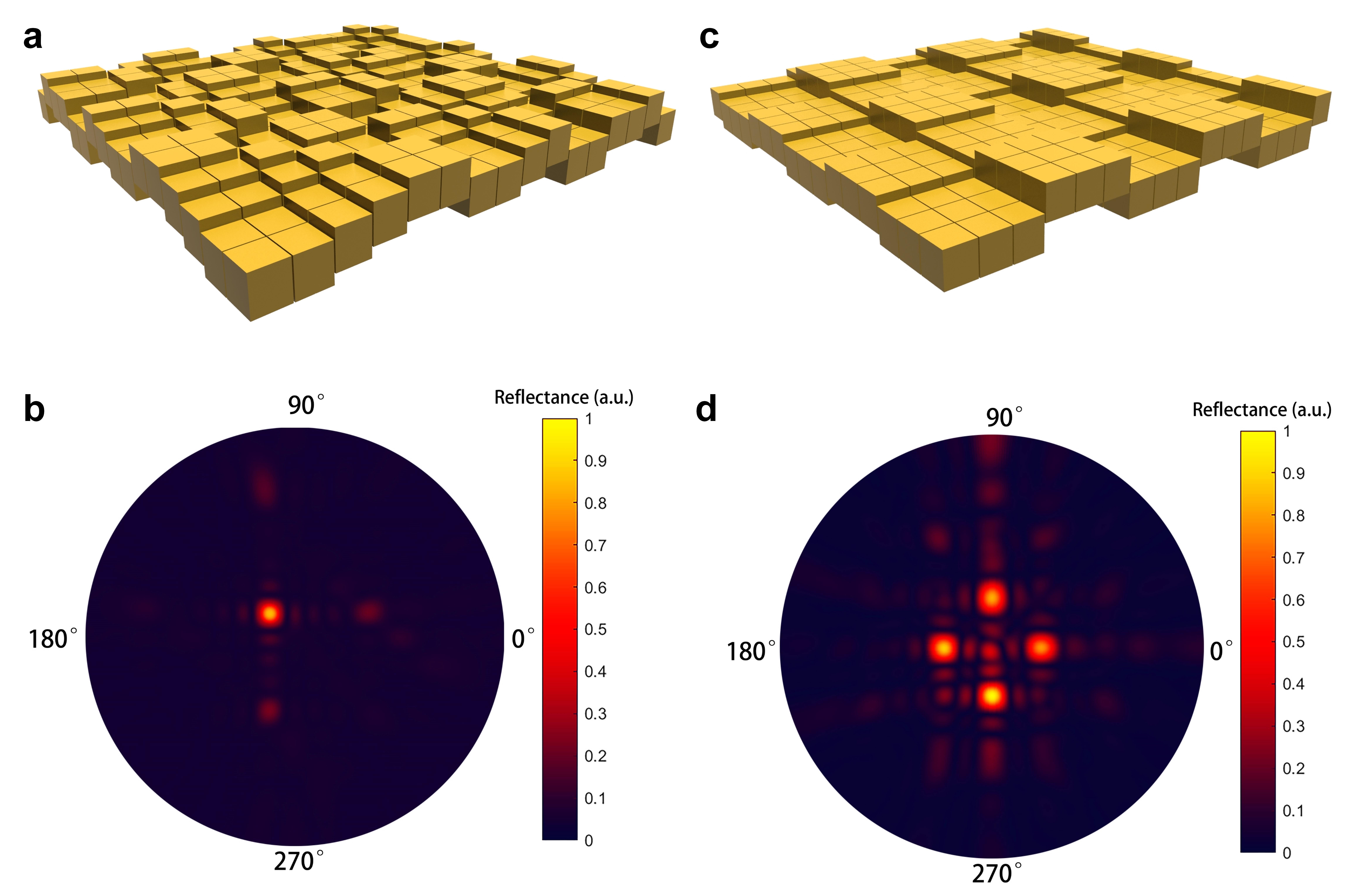


**Supporting Information Figure S8** Two examples to demonstrate the beam shaping performance. a) Coding pattern to defect the normal incidence to the direction of (27.1°,135°), obtained by multiplexing a “00 01 10 11 00 01 10 11” (super unit cell size N=2) with a “00 01 10 11 00 01 10 11” (super unit cell size =3) in the x direction and add a same coding sequences in the y direction. b) Simulated 3-D radiation patter *N* n for the radiation pattern in (a). c) Coding pattern to split the normal incidence to four directions of (20.1°, 0°), (20.1°, 90°), (20.1°, 180°), (20.1°, 270°), obtained with a matrix [00 01; 11 10] (super unit cell size *N*=3). (d) Simulated 3-D radiation pattern for the radiation pattern in (c).


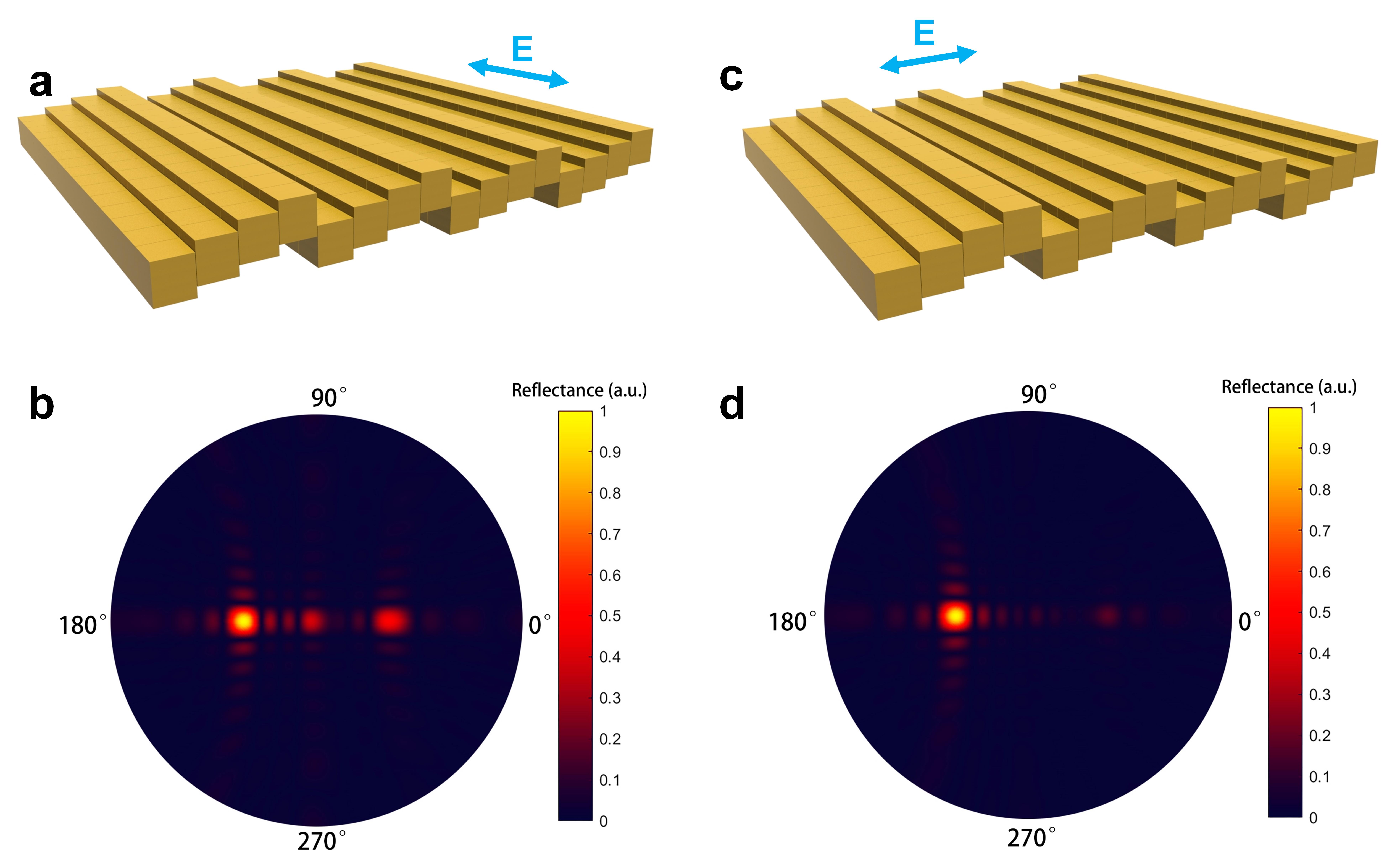


**Supporting Information Figure** **S9** Two examples to investigate the influence of the height difference on the radiation patterns. a,c) Coding pattern “00 01 10 11 00 01 10 11…” (super unit cell size *N*=1). b,d) Simulated 3-D radiation pattern for the radiation pattern in (a) when the incident beam is polarized b) perpendicular, and d) parallel to the varying direction of coding sequence. Both cases show a single radiation beam in the direction of (32.4°,180°).


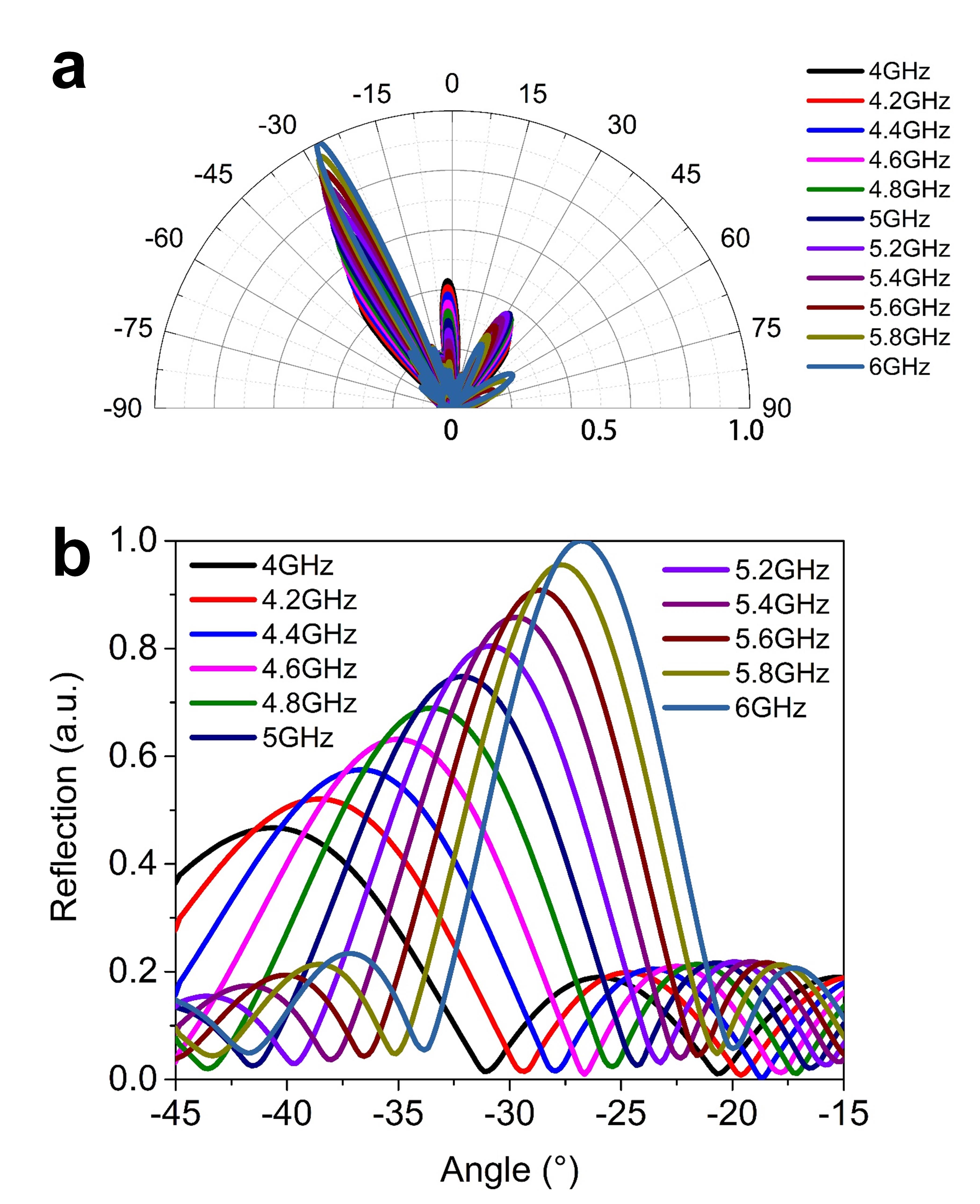


**Supporting Information Figure S10** Simulated radiation pattern of only the phase coding pattern “00 01 10 11 00 01 10 11…” (super unit cell size *N*=1), without the group delay coding pattern. a,b) Simulated radiation pattern in the polar coordinate and Cartesian coordinate, respectively.


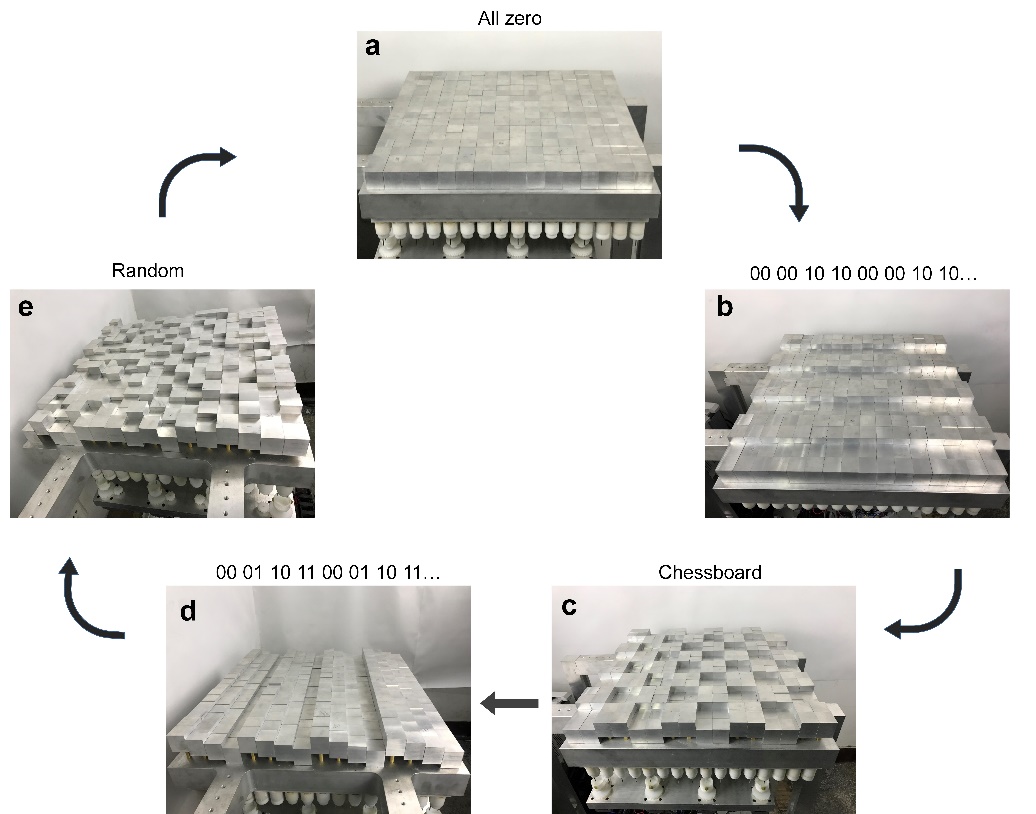


**Supporting Information Figure S11** The entire process of the MCPM prototype in switching among a series of coding patterns. a) All zero. Video V2. b) “00 00 10 10 00 00 10 10...”. Video V3. c) Chessboard (super unit cell size N=2). Video V4. d) “00 01 10 11 00 01 10 11…”. Video V5. e) Random. Video V6.
